# The employment of real-time polymerase chain reaction for analysis of canine meat in meatball products for halal authentication analysis

**DOI:** 10.5455/javar.2024.k770

**Published:** 2024-06-04

**Authors:** Rumiyati Rumiyati, Rien Larasati Arini, Purwanto Purwanto, Abdul Rohman

**Affiliations:** 1 Faculty of Pharmacy, Universitas Gadjah Mada, Yogyakarta, Indonesia; 2 Center of Excellence, Institute of Halal Industry and Systems (IHIS), Universitas Gadjah Mada, Yogyakarta, Indonesia

**Keywords:** Canine meat, meatball, species-specific primers, real-time PCR, halal certification

## Abstract

**Objective:**

Meatballs are a popular meat-based food consumed widely in Indonesian society. However, the issue of unethical substitution of halal meatballs with non-halal meats, particularly pork and canine meat (CM), has emerged. The existence of non-halal meats, including CM, in food products is prohibited in Islam, necessitating the development of reliable analytical techniques for their identification. In this study, we designed species-specific primers (SSPs) targeting the D-loop region of mitochondrial DNA for CM meatball product identification.

**Materials and Methods:**

The study was commenced by creating specific primers for canine DNA using Integrated DNA Technologies software and subsequently performing DNA isolation. The designed primers were then subjected to comprehensive evaluation using RT-PCR, including specification, linearity, limit of detection, efficiency, and repeatability.

**Results:**

The results indicated that the primer D-Loop 443 (forward: 5’-GGG ACA TCT CGA TGG ACTA ATG-3’, reverse: 5’-GCG GTC ATA GAT GAG TGA TAG C-3’) designed and validated in silico using primer-basic local alignment search tool nucleotide (BLAST) program from NCBI accurately identified canine DNA when the optimal annealing temperature was set at 57.5^o^C. The real-time PCR technique utilizing the D-loop 443 primer exhibited the ability to amplify canine DNA down to a minimum quantity of 100 pg, with an efficiency value of 91.8%, a correlation coefficient (R) of 0.990, and a precision value (RSD) of 0.30%.

**Conclusion:**

The SSP-based RT-PCR method developed is a versatile and efficient tool for detecting CM in meatballs. Its implementation helps maintain consumer trust and addresses concerns regarding the substitution of halal meats with non-halal alternatives.

## Introduction

Meatballs are a highly popular meat-based food that is frequently consumed by Indonesian society. Meat is considered a critical component in food products due to the possibility of it being sourced from non-halal animals or halal animals that are not slaughtered in line with Syariah principles [1]. The utilization of non-halal foods, such as pig, rat, and canine meat (CM), is an emerging issue in Indonesia, and some cases have been reported where unethical sellers have replaced or mixed halal meatballs with non-halal meat [2].

The most reported ones are the substitution of beef meatballs with pork. The motivation underlying this practice is the economic reason why beef meatballs have a higher price than pork meatballs. CM as non-halal meat may be a potential adulterant in beef meatballs since stray canines can be obtained freely. The issue of consuming food that includes dog meat is highly contentious, particularly in nations where the practice has been eradicated for a significant period or has rarely or never occurred [3]. However, CM may be consumed and used as food components in countries such as China, South Korea, and Vietnam [4]. The presence of CM in any product is prohibited by Muslims; therefore, some analytical tools that can accurately identify non-halal meats, including CM, are urgent. Even more, Indonesia has mandated that all food products supplied and sold inside its borders must possess a halal certification. The products that are believed to include non-halal meats should undergo laboratory testing [5].

During the halal authentication analysis of meats, it is necessary to utilize validated and established methods. Some analytical methods based on the analysis of specific markers, fingerprinting profiling, and metabolomics approaches have been suggested and utilized for the examination of non-halal meats. The two-dimensional GC, coupled with mass spectrometry, is a highly successful method for identifying non-halal meats. It achieves this by detecting certain fatty acid markers [6]. Unfortunately, this method involves advanced instruments with expensive investment; therefore, it is not suitable for routine analysis in halal meat authentication. Fingerprint profiling based on spectroscopic techniques has also been reported, including Fourier transform infrared (FTIR) spectroscopy [7–11], Raman spectroscopy [12,13], and NMR spectroscopy [14]. However, using sophisticated data analysis to treat the data coming from these molecular spectra is unavoidable. In recent years, the utilization of metabolomics methodologies using high-resolution liquid chromatography-mass spectrometry has been a potential technology for the detection and identification of non-halal meats; however, the metabolites obtained are very large, which makes the data interpretation difficult [15]. For this reason, DNA-based methods are the preferred technique for analyzing non-halal meats due to their specificity and sensitivity [16,17,18].

Real-time polymerase chain reaction (RT-PCR) is widely considered the most reliable method for identifying DNA in animal-derived nutritional products. It offers both high specificity and sensitivity, as each type of meat has its own distinct DNA. RT-PCR enables the accurate and efficient measurement of DNA in food products [19]. RT-PCR is a preferred approach for diagnosing non-halal meats because of its specificity and reproducibility, making it advantageous for identifying reasons [18]. The methods have been developed as a tool for meat authentication in some food products, e.g., pork and chicken in sausage products [20], pork in meatballs [21], bovine in gummy candy [22], and pork and wild boar in chicken sausages [23]. Previous research has used a variety of primer sources of DNA extracted from meat samples. The species-specific primers (SSPs) focusing on Mitochondrial Cytochrome b have been applied in the studies for the identification of CMballs [24], dog meatballs [4], and wild boar meatballs [25,26], respectively. The use of SSP that targets D-loop mitochondria for authentication of wild boar in meatballs was done by Arini et al. [25]. However, SPP that was targeted to the mitochondrial D-loop for authentication of CM in meatballs has not been done yet. Therefore, the objective of this study was to develop SSP targeting in the D-loop region for detecting CM in meatball products. The designed primers were further validated and implemented for the analysis of meatball products in the local markets.

## Materials and Methods

### Primer designing

Five sets of primers targeting the D-loop fragment of a canine’s mitochondrial DNA The design was created utilizing software provided by Integrated DNA Technologies (IDT, USA). The DNA sequences of *Canis familiaris* mitochondrial DNA were acquired from NCBI (http://www.ncbi.nlm.nih.gov). A set of primers was chosen from the five candidates after performing the specificity assessment *in silico*, namely basic local alignment search tool nucleotide (BLASTn). In the *in silico* test, the presence of predicted secondary structures was determined by the Oligo Analyzer Tool from IDT software.

### DNA extraction

DNA from raw meats was isolated using FavorPrepTM Tissue Genomic DNA Extraction Mini Kit manufactured by Favorgen in Taiwan. The procedure involved several processes, namely cell lysis, protein degradation, DNA binding with a silica matrix, washing away contaminants, and eluting DNA from the silica matrix. These steps were carried out according to the instructions provided by the manufacturer. The DNA isolate was analyzed qualitatively and quantitatively using NanoQuant Spark Tecan (Switzerland) at wavelengths of 260 and 280 nm to ascertain the concentration and purity of DNA. Isolated DNA is deemed pure if the A260/A280 ratio falls within the range of 1.8–2.0.

### Real-time PCR analysis

The targeted DNA was amplified using PCR CFX96 (Biorad, USA) with a total volume of 10 µl. This included 5 µl of 2x SensiFast SYBR No-Rox Kit (Meridian Bioscience, USA), 0.4 µl of forward primer, and 0.4 µl of reverse primer (final concentration 0.4 µM), 1 µl of DNA with a concentration of 10 ng/µl, and 3.2 µl of nuclease-free water. Pre-denaturation was done at a temperature of 95°C as long as 3 min, then denaturation (25 cycles) was continued at the same temperature (95°C) for 5 sec. The annealing temperature varied from 51.2°C to 61.2°C, with a duration of 10 sec at each temperature. The elongation phase was conducted for 20 sec at a temperature of 72°C.

### Validation of qPCR-SSP

The designed primer was confirmed through the assessment of characteristics including specificity, linearity, efficiency value, the limit of detection (LoD), and repeatability. The acceptance criteria for each parameter were based on Codex Alimentarius. The method that was confirmed to be accurate and reliable was then employed for the analysis of meatball products commercially available around Yogyakarta, Indonesia.

## Results and Discussion

The standard approach for determining the presence of non-halal meats in food products is a DNA-based method using qPCR because of its specificity. The goal of this work was to develop a real-time PCR technique using a brand-new SSP to identify canin in processed foods. The primer was made to specifically target a D-loop segment from *C. familiaris*’s mitochondrial DNA. The D-loop segment was selected as the targeted DNA due to its rapid evolutionary rate and the abundance of mitochondrial DNA. This makes it suitable for species identification and enhances the specificity of the primer that was designed [27,28]. Using nucleotide BLAST from the NCBI database, five primer candidates were put through an in silico specificity test. Results are shown in Table 1.

**Table 1.** Primer D-loop 443 targeting D-loop fragment on mitochondrial DNA of *C. familiaris.*

Primer sequence 5‘–3‘	Tm (°C)	GC content (%)	Amplicon length
GGGACATCTCGATGGACTAATG	62	50	118-bp
GCGGTCATAGATGAGTGATAGC	62	50	

**Table 2.** Concentration and purity evaluation of isolated DNA from raw meat and commercial meatball samples.

Samples	DNA concentration (ng/µl)	Purity index (A260/A280)
Canine	182.47	1.96
Chicken	85.44	1.93
Pork	137.66	1.92
Wild Boar	186.97	2.07
Goat	154.57	1.92
Beef	60.57	1.96
Rat	75.49	1.94
Meatball 1	101.19	1.90
Meatball 2	86.13	2.08
Meatball 3	57.13	2.03
Meatball 4	79.61	1.95
Meatball 5	204.36	1.98
Meatball 6	82.45	2.00
Meatball 7	101.91	2.06
Meatball 8	34.60	1.91
Meatball 9	58.08	1.90
Meatball 10	344.55	1.87
Meatball 11	590.94	1.89
Meatball 12	607.39	1.89

The DNA was isolated from raw meat using a silica-based method. The extracted DNA was quantified by measuring its absorbance at 260 nm, while its quality was assessed by calculating the absorbance ratio at 260 and 280 nm (A260/A280). The ideal A260/A280 ratio for pure DNA is 1.8–2.0, making it suitable for use as a DNA template for real-time PCR amplification. Table 2 displays the purity and concentration of DNA. The results are shown in Table 2.

**Figure 1.**
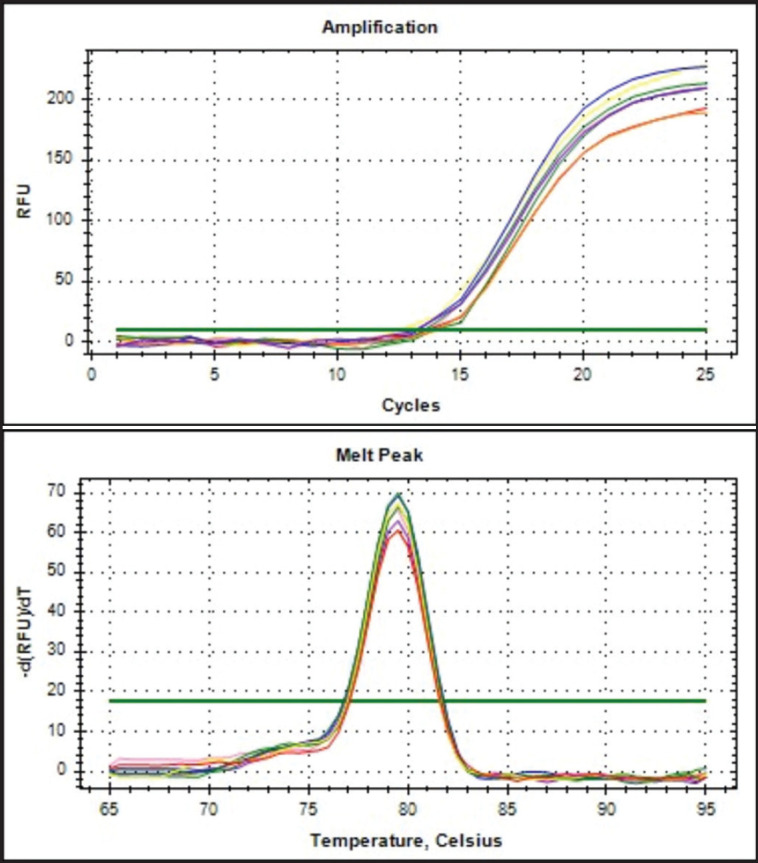
Amplification curve of annealing temperature optimization at different temperatures and optimum at 57.5°C.

The annealing temperature (Ta) was optimized around the calculated Ta range before performing real-time PCR parameter validation. Ta can be predicted using the primer’s melting temperature (Tm). Ta is typically 3°C–5°C lower than Tm. The optimization of Ta was done within the temperature range of 51.2°C to 61.2°C. The annealing temperature of 57.5°C was selected due to its ability to generate the greatest intensity at the lowest quantification cycle (Cq) value, as demonstrated in Figure 1. This temperature will be utilized in the subsequent validation operation.

**Figure 2.**
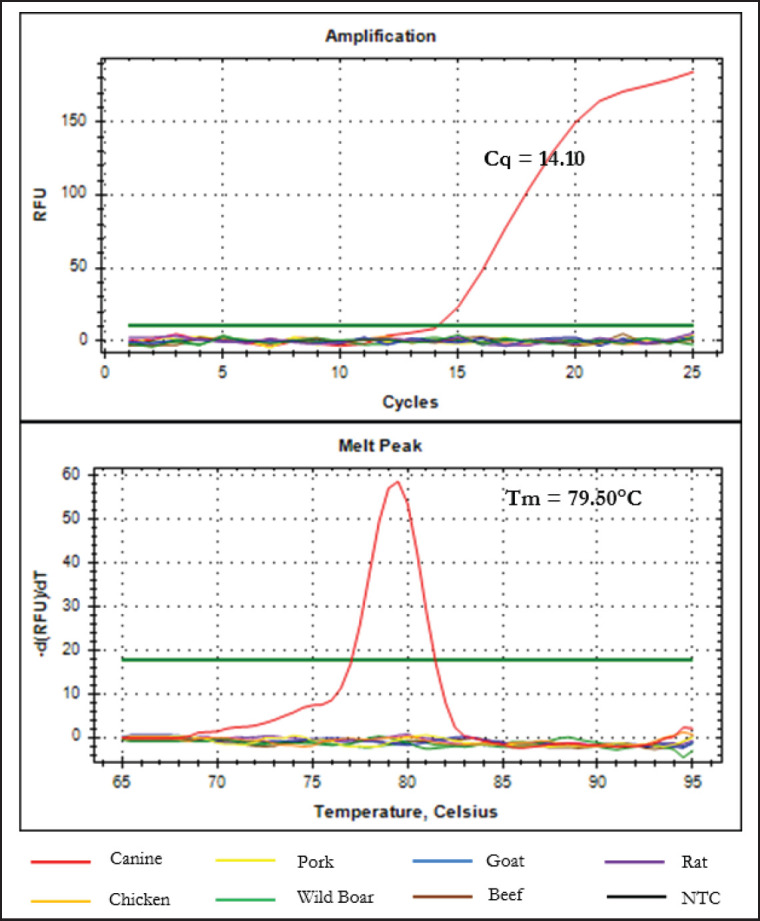
The specificity test of primer D-Loop 443 targeting D-Loop fragment of mitochondrial DNA of CM tested with 6 other species sequences. (A) Amplification curves; (B) Melting peak.

To test the specificity of the designed primer, DNA from eight other species was isolated: canine (*C. familiaris*), chicken (*Gallus gallus*), pork (*Sus scrofa domesticus*), wild boar (*Sus scrofa*), goat (*Capra hircus*), beef (*Bos taurus*), and rat (*Rattus novergicus)*. The specificity test demonstrates that the primer could only amplify canine DNA and not the DNA of six other species, as shown in Figure 2. The melt peak has a sharp peak, indicating that no secondary structure was formed during amplification.

**Figure 3.**
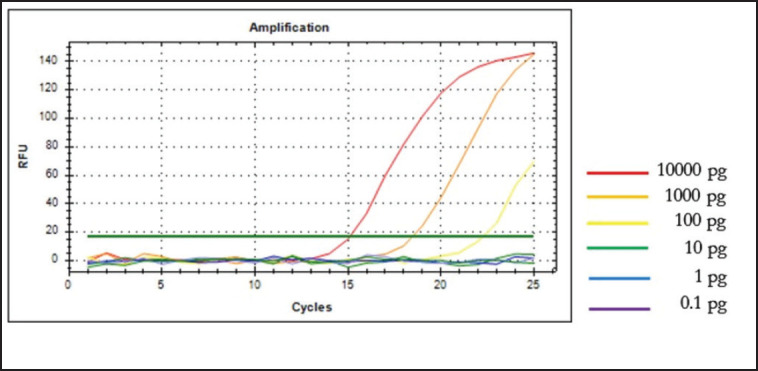
Amplification of canine‘s DNA at different DNA concentrations for sensitivity test using D-loop 443 primer of *C. familiaris*.

Sensitivity was assessed by performing a 10-fold serial dilution of canine DNA in the range of 10,000–0.1 pg, followed by establishing the precise threshold of detection (LoD). According to the amplification curve, the LoD value for detecting CM DNA was 100 pg, the lowest concentration that yields an amplification curve. No amplification curves were detected when the DNA concentration was below 100 pg, as shown in Figure 3.

**Figure 4.**
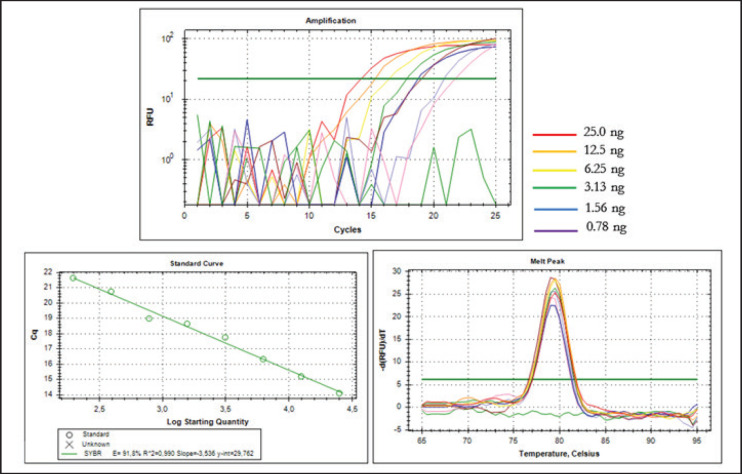
Amplification using the primer D-loop 443 with serial dilution of DNA template for efficiency analysis (A) Amplification curves; (B) Standard curves; (C) Melting peak.

The efficiency of the PCR methods was evaluated by performing a two-fold serial dilution of the CM DNA. A standard curve was generated by plotting the quantity of DNA against the Cq value for each concentration. This was done to determine the efficiency (E) value. A precise linear regression was obtained from the standard curve of the amplification response, as depicted in Figure 4. The correlation coefficient (*R*^2^) value was 0.990, while the slope and intercept were -3.536 and 29.762, respectively. The *R*^2^ value is =0.98, which means that the curve satisfies the sufficiently good linearity criteria. Furthermore, a calculation of the PCR efficiency value (E) utilizing the equation E = [10(-1/slope)-1] was 91.8%, which was considered to be good because the E value was within the suggested range (90%–110%) of qPCR efficiency [29].

**Figure 5.**
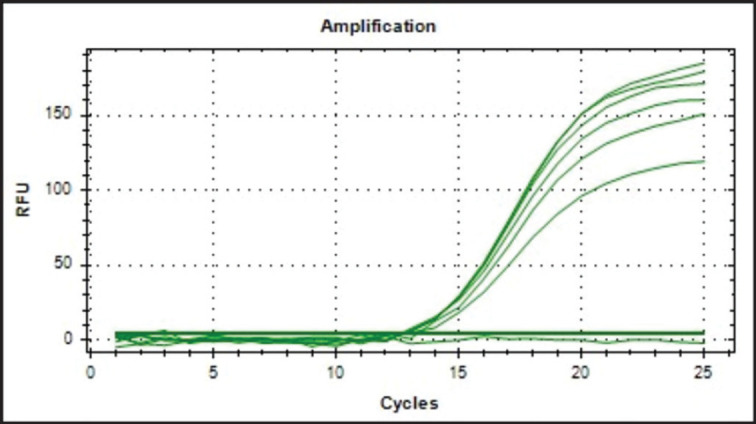
Amplification using primer D-loop 443 for repeatability analysis.

**Figure 6.**
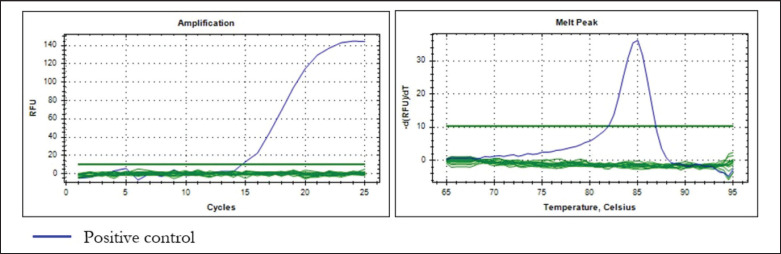
D-loop 443 fragment DNA amplification was performed on 12 commercial meatball samples.

Figure 5 displays the fluctuations in PCR amplicon Tm observed in our test. The quantitative PCR analysis was conducted using 10 nanograms of canine DNA. The resulting Cq values were 12.68, 12.48, 12.66, 13.27, 12.59, and 12.40. The CV for the repeatability of raw beef canine DNA was 0.30%, indicating a high level of reproducibility for the method that was devised. The CV values of canine DNA have satisfied the recommended standards for the use of the PCR method (CV = 25%) [29,30].

The validated qPCR method with primer D-loop 443 was used to detect CM in 12 commercial meatball samples collected in Yogyakarta, including a negative control (NTC) with no template and a positive control (containing isolated DNA from beef). The results show that there is no amplification observed from 12 meatball samples and NTC, indicating that the meatball samples are negative for CM, whereas the positive control exhibits amplification with a Cq value of 14.76, as shown in Figure 6. In summary, the results demonstrate that the developed method exhibits a low LoD, specificity, high precision, and satisfactory efficiency, rendering it suitable for the analysis of commercial meatball samples.

## Conclusion

Mitochondrial D-loop 443 primers can detect the existence of canine DNA in meat. The primers possess an optimal annealing temperature of 57.5°C, and the developed method can increase the quantity of DNA with a LoD of 100 pg of DNA. It has a high replicability with an efficiency value of 91.8%, an acceptable correlation (*R* of 0.990), and an acceptable precision (CV of 0.30%). It has the potential to be implemented in authentication meat in CMballs.

## List of Abbreviations

BLAST, basic local alignment search tool; CM, canine meat; Cq, quantification cycle; Ct, threshold cycle; CV, coefficient of variation; DNA, deoxyribonucleic acid; FTIR, Fourier transform-infra red; Lod, Limit of Detection; NCBI, National Center for Biotechnology Information; NMR, nuclear magnetic resonance; PCR, polymerase chain reaction; qPCR, Quantitative PCR; SSP, species-specific primers; Ta, annealing temperature; Tm, melting temperature.
